# Smart Nanoparticles Based on Hyaluronic Acid for Redox-Responsive and CD44 Receptor-Mediated Targeting of Tumor

**DOI:** 10.1186/s11671-015-0981-5

**Published:** 2015-07-11

**Authors:** Hyung-Kyu Park, Sang Joon Lee, Jong-Suk Oh, Sam-Gyu Lee, Young-IL Jeong, Hyun Chul Lee

**Affiliations:** Department of Microbiology, Chonnam National University Medical School, 160, Baekseo-ro, Dong-gu, Gwangju, 501-746 Republic of Korea; Department of Biomedical Sciences, Chonnam National University Medical School, Gwangju, 501-746 Republic of Korea; Department of Physical and Rehabilitation Medicine, Chonnam National University Medical School, Gwangju, 501-746 Republic of Korea; Biomedical Research Institute, Pusan National University Hospital, Pusan, 602-739 Republic of Korea

**Keywords:** Hyaluronic acid, CD44 receptor-mediated endocytosis, Redox-responsive, Block copolymer, Nanoparticles

## Abstract

**Background:**

Since aggressive cancer cells highly express the CD44 receptor compared to normal cells, hyaluronic acid (HA) can be used for CD44 targeting molecule. Since glutathione (GSH) level is normally elevated in the intracellular compartment and in the tumor cell, the fact that disulfide bond can be cleaved by GSH is widely used for intracellular drug delivery.

**Methods:**

HA was connected with poly(dl-lactide-co-glycolide) (PLGA) using disulfide linkage, and then a diblock copolymer (HAssLG) was prepared. Doxorubicin (DOX)-loaded HAssLG nanoparticles were prepared by dialysis procedures.

**Results and Discussion:**

DOX-loaded HAssLG nanoparticles have spherical shapes with small particle size of less than 300 nm. In fluorescence measurement, DOX was dose-dependently liberated from nanoparticles by the addition of GSH. DOX release rate from HAssLG nanoparticles was increased by the addition of GSH. To confirm CD44 receptor-mediated endocytosis of nanoparticles, CD44-positive MDA-MB231 cells were employed and fluorescence intensity was strong when nanoparticles were treated to tumor cells. However, fluorescence intensity was significantly decreased through blocking of the CD44 receptor by pretreatment of cells with free HA. Fluorescence intensity of cells was increased again when GSH was added, indicating that HAssLG nanoparticles have CD44 receptor targetability and potential of redox-responsive drug delivery. For animal imaging study, CD44-positive MDA-MB231 cells and CD44-negative NIH3T3 cells were simultaneously implanted into the right flank and left flank of mice, respectively. Fluorescence intensity was significantly stronger at tumor mass of MDA-MB231 cells than solid mass of NIH3T3 cells, indicating that HAssLG nanoparticles were specifically delivered to tumor cells.

**Conclusions:**

The results indicated that HAssLG nanoparticles have specificity against the CD44 receptor and can be used for anticancer drug targeting. We recommend HAssLG nanoparticles as a promising vehicle for cancer drug targeting.

## Background

Nanomedicines based on nanotechnology have been extensively investigated for drug delivery issues, and they can be considered as appropriate vehicles for tumor targeting due to their small sizes [1–4]. Especially, nanosized vehicles such as nanoparticles or polymeric micelles have superior potentials for solid tumor targeting because the enhanced permeation and retention (EPR) effect against tumor blood vessels is regarded as a preferential advantage of nanovehicles for solid tumor targeting [5, 6]. Furthermore, nanoparticles can be decorated with various targeting moieties for specific targeting of cancer cells since they have a big surface area [7–10]. Due to these intrinsic properties, nanoparticles can concentrate cytotoxic anticancer drug to the solid tumor with minimal unwanted side effects for normal tissues/cells. From these points of view, nanoparticles decorated with targeting moieties have been also extensively investigated [7–10].

Aggressive tumor cells frequently overexpress the CD44 receptor in the cell membrane [11–18]. This is a specific receptor for hyaluronic acid (HA), and HA is known to stimulate spreading, migration, invasion, and metastasis of cancer cells [11–14, 17]. From these reasons, hyaluronic acid has been extensively used as a vehicle for specific targeting of the CD44 receptor of tumor cells [8, 18–22]. Especially, Prestwich’s group extensively investigated the potential of CD44 receptor delivery of HA-anticancer agent conjugates against cancer cells [19–22]. Furthermore, HA was also investigated for tissue engineering, gene delivery, and peptide delivery due to the biodegradability and biocompatibility [23–25].

Nanoparticles based on redox-responsive drug targeting for cancer cells also have been extensively investigated in the last decades [26–29]. Redox-responsive drug delivery is based on the fact that disulfide bond can be reduced by glutathione and intracellular glutathione (GSH) level is significantly higher than the extracellular fluid level [30]. This peculiar property between GSH and disulfide bond stimulated the redox-responsive drug delivery concept. For example, Sun et al. synthesized a poly(ethylene glycol)/poly(ε-caprolactone) block copolymer using disulfide bond [27]. They showed that drug release rate was increased by the addition of dithiothreitol (DTT) and enhanced tumor cell uptake of anticancer drugs was observed. Furthermore, redox-responsive delivery of DNA drugs using disulfide-linked polymer nanovehicles was also investigated by several authors [26–29]. In spite of successful development of the redox-responsive drug delivery system, GSH levels in normal tissues and low specificity of nanoparticles are still obstacles for efficient drug targeting. For example, GSH levels can be increased in the tissues of a tumor-bearing mice compared to a normal mice [30]. Therefore, multiple targeting strategies are required to attain efficient targeting of tumors.

In this study, we synthesized a HA-*b*-PLGA (HAssLG) copolymer having disulfide linkage between HA and PLGA for targeting of tumor cells through the CD44 receptor and redox-responsive process. Since PLGA is a biodegradable and lipophilic polymer, PLGA must form the core of the nanoparticles while HA would form the outer shell of the nanoparticles. We studied the potential of CD44 receptor-mediated and redox-responsive targeting of tumor cells by HAssLG nanoparticles using tumor cells and fibroblast cells in vitro and in vivo.

## Methods

### Materials

HA (molecular weight from the manufacturer’s data, 7460 g/mol) was purchased from Lifecore Biomedical (Chaska, MN, USA). Triethylamine (TEA), L-glutathione (GSH), sodium cyanoborohydride, *N*-(3-dimethylaminopropyl)-*N*′-ethylcarbodiimide hydrochloride, and cystamine hydrochloride were purchased from Sigma Chem. Co. (St. Louis, USA). *N*,*N*′-dicyclohexyl carbodiimide (DCC) and *N*-hydroxysuccimide (NHS) were purchased from Sigma-Aldrich Chemical Co, USA. The dialysis membranes (Spectra/Pro™ membranes) with molecular weight cutoffs (MWCO) of 2000 and 12,000 g/mol were purchased from Spectrum Labs. Doxorubicin (DOX) HCl was purchased from LC Labs Co. Ltd. (Woburn, MA, USA). Dichloromethane (DCM) and dimethyl sulfoxide (DMSO) were of HPLC grade or extra-pure grade. PLGA (PLGA-5005, MW = 5000 g/mol) was purchased from Wako Pure Chemicals Co. (Osaka, Japan). The molecular weight (MW) of PLGA was measured by GPC, as described previously [8]. The weight average MW, number average MW, and polydispersity of PLGA were 4920, 4780, and 1.029, respectively.

### Synthesis of HAssLG Block Copolymer

HA having disulfide linkage in the end of the chain was synthesized as described by Maruyama et al. [25] with brief modification. HA (400 mg) was dissolved in 10 ml of H_2_O/DMSO (3/7, *v*/*v*) solution, and then excess amount of sodium cyanoborohydride was added to this solution followed by magnetic stirring for 12 h at room temperature. After that, 10 equivalents of cystamine were added and the mixtures were further stirred for 24 h. The resulting solution was put into a dialysis membrane (MWCO, 2000 g/mol), and by-products were removed by dialysis procedure for 2 days followed by lyophilization for 3 days.

NHS-activated PLGA (PLGA-NHS) was separately prepared. Five hundred milligrams of PLGA was dissolved in DCM with 1.2 equivalents of DCC and NHS. This solution was stirred for 12 h, and then reactants were precipitated into the excess amount of methanol followed by filtration under reduced pressure. The white solid was washed with methanol three times followed by vacuum drying for a day.

The HAssLG block copolymer was synthesized as follows: 400 mg HA-cystamine conjugates and 250 mg of PLGA-NHS were simultaneously dissolved in dry DMSO and stirred in nitrogen atmosphere for 2 days. After that, this solution was then introduced into a dialysis membrane (MWCO, 12,000 g/mol) to remove the unreacted HA and organic solvent. During the dialysis procedure, water was exchanged at 3-h intervals for 2 days and then the resulting solution was lyophilized for 3 days. The lyophilized solid was precipitated into DCM to remove unreacted PLGA for three times, and then filtered precipitates were dried under a vacuum for 3 days. The yield of the final product was 81 % (*w*/*w*). Yield of product (%, *w*/*w*) = [(weight of final product)/(weight of aminated HA + PLGA-NHS)] × 100.

### ^1^H Nuclear Magnetic Resonance Spectroscopy

^1^H nuclear magnetic resonance (NMR) spectroscopy (500 MHz superconducting FT-NMR spectrometer, Unity-Inova 500, Varian Inc., Agilent Tech., CA, USA) was employed to confirm synthesis of polymers.

### Preparation of HAssLG Nanoparticles

Empty or DOX-incorporated nanoparticles were fabricated as follows (Table 1): 50 mg of HAssLG block copolymer was dispersed in 1 ml of deionized water and then mixed with 3 ml DMSO. DOX·HCl was separately dissolved in 1 ml DMSO with trace amounts of TEA, and then this solution was added to the polymer/DMSO solution. Then, the mixed solution was dialyzed against deionized water using a dialysis membrane (2000 g/mol) with exchange of water (2-h intervals) for 1 day. After that, the resulting solution was analyzed or lyophilized. Empty nanoparticles were fabricated except DOX. To measure DOX contents in the HAssLG nanoparticles, 5 mg of DOX-loaded nanoparticles was dissolved in 10 ml of DMSO/water mixtures (8/2, *v*/*v*) and then appropriately diluted with DMSO. The DOX contents in the nanoparticles were evaluated with a UV spectrophotometer (UV spectrophotometer 1601, Shimadzu Co., Japan) at 486 nm. To avoid interference of the polymer, empty HAssLG nanoparticles were used as blank. Drug contents were calculated with the following equation: [(drug weight in the nanoparticle)/(total weight of the nanoparticle)] × 100.

**Table 1:** Characterization of HAssLG nanoparticles

Polymer/drug (mg/mg)	Drug contents (%, *w*/*w*)	
	Theoretical	Experimental
50/0		
50/4	7.4	4.3
50/7	12.3	7.8

### Analysis of Nanoparticles

Transmission electron microscopy (TEM, JEOL JEM-2000 FX II, Japan) was used to observe the morphology of nanoparticles. One drop of nanoparticle solution (0.1 mg/ml) was dropped onto a carbon film coated on a copper grid and then dried in room temperature. Three hours later, phosphotungstic acid (0.05 %, *w*/*w*) was then dropped onto nanoparticles for negative staining and dried in room temperature for more than 6 h. Observation of nanoparticles was performed at 80 kV.

Particle size was measured with dynamic laser scattering (DLS-7000, Otsuka Electronics Co., Japan) (nanoparticle concentration, 0.1~1 mg/ml).

### Drug Release Study

DOX release from HAssLG nanoparticles was performed as follows: 10 mg of DOX-incorporated nanoparticles was dispersed in 5 ml of phosphate-buffered saline (PBS, 0.1 M, pH 7.4), and this solution was put into a dialysis membrane (MWCO, 12,000 g/mol). After that, the dialysis membrane was introduced into 95 ml PBS in a bottle. Drug release was performed in a shaking incubator with 100 rpm of stirring speed at 37 °C. Whole media were replaced with fresh media at specific time intervals. The released DOX was measured with a UV spectrophotometer (UV spectrophotometer 1601, Shimadzu Co., Japan) at 486 nm.

### Cell Culture

MDA-MB231 human breast carcinoma cells and NIH3T3 mouse fibroblast cells were obtained from Korea Cell Line Bank Co. (KCLB, Seoul, Korea). MDA-MB231 human breast carcinoma cells and NIH3T3 mouse fibroblast cells were maintained in RPMI1640 and DMEM supplemented with 10 % fetal bovine serum (5 % CO_2_ at 37 °C), respectively.

Cell viability test was performed as follows: MDA-MB231 (1 × 10^4^ cell/well) cells were seeded into 96-well plates and then incubated overnight (5 % CO_2_ at 37 °C). DOX or nanoparticles were treated to these cells. The CD44 receptor of the cells was blocked with 10 equivalents of free HA 1 h before nanoparticle treatment, and then the cells were washed with PBS. After that, DOX or nanoparticles were added to the cells in the 96-well plates and incubated in a CO_2_ incubator (5 % CO_2_ at 37 °C) for 4 or 24 h. After that, media were discarded and then replaced with 100 μl of fresh media. To this solution, 30 μl of MTT (5 mg/ml) was added, and then the solution was further incubated in a CO_2_ incubator (5 % CO_2_ at 37 °C) for 4 h. Since the contents of the generated formazan were proportional to the number of viable cells, the generated formazan was solubilized with DMSO and determined with an automated computer-linked microplate reader (Molecular Device Co., USA) (560 nm test/630 nm reference).

### Receptor-Mediated Delivery of Nanoparticles to Tumor Cells

Cellular uptake of DOX-loaded HAssLG nanoparticles was studied with fluorescence microscopy observation and flow cytometry analysis. MDA-MB231 cells were seeded onto a cover glass and incubated overnight in a CO_2_ incubator (5 % CO_2_ at 37 °C). Then, nanoparticles were treated to cells for 1 h in a CO_2_ incubator. One hour before the addition of nanoparticles, the CD44 receptor was blocked by pretreatment of free HA (10 equivalents of nanoparticle contents). Following this, cells were washed with PBS (pH 7.4, 0.1 M) and fixed with 4 % paraformaldehyde. This was fixed with immobilization solution (IMMU-MOUNT, Thermo Electron Corporation, Pittsburgh, PA, USA). Cells were then observed with a confocal laser scanning microscope (CLSM, TCS-SP2; Leica, Wetzlar, Germany).

For flow cytometry analysis of cells, 1 × 10^6^ cells were seeded in each well of six-well plates. Nanoparticles were treated to the cells and incubated for 1 h in a CO_2_ incubator. One hour before the addition of nanoparticles, the CD44 receptor was blocked by pretreatment of free HA (10 equivalents of nanoparticle contents). Cells were washed with PBS (pH 7.4, 0.1 M) and trypsinized to harvest cells. The cells were analyzed with flow cytometery (FACScan) at an excitation wavelength of 488 nm and emission wavelength of 522 nm for DOX fluorescence intensity.

### Near-Infrared Absorption Dye Conjugation

A near-infrared (NIR) dye was conjugated with HAssLG for in vivo animal imaging studies. Fifty milligrams of HAssLG in 5 ml DMSO/water mixtures (4/1, *v*/*v*) was mixed with 3 mg of sulfo-cyanine7 amine (Lumiprobe Co., Florida, USA) with 1.2 equivalent moles of EDAC and NHS. This solution was then stirred for 24 h in room temperature. After that, the solution was dialyzed to remove unreacted dye with exchange of water at intervals of 2 h until the dye was not detected in the water. The detection of the dye and the contents in the polymer were measured with a UV spectrophotometer (UV-1601 UV-VIS spectrophotometer, Shimadzu, Kyoto, Japan) at 750 nm. The content of the NIR dye was 2.8 %, *w*/*w*. (Dye content (%, *w*/*w*) = (dye amount in the nanoparticles/total weight of the nanoparticles) × 100).

### In Vivo Fluorescence Imaging

For fluorescence imaging of the biodistribution of nanoparticles, 1 × 10^6^ MDA-MB231 cells and 1 × 10^6^ NIH3T3 cells were implanted into the right flank and left flank of nude BALb/C mice (5 weeks, 20 g), respectively. After 20 days, NIR-dye-conjugated nanoparticles were administered intravenously (i.v.) into the tail vein of mice at a dose of 20 mg/kg (injection volume was 100 μl). Mice were observed with a Maestro™ 2 small-animal imaging instrument (Cambridge Research and Instruments, Inc., Woburn, MA, USA). Twenty-four hours later, mice were sacrificed to evaluate organ distribution of nanoparticles with the Maestro™ 2 small-animal imaging instrument. All animal studies were performed under the guidelines of the Committee of Care and Use of Laboratory Animals of Chonnam National University.

## Results

### Characterization of HAssLG Block Copolymer

**Fig. 1:**
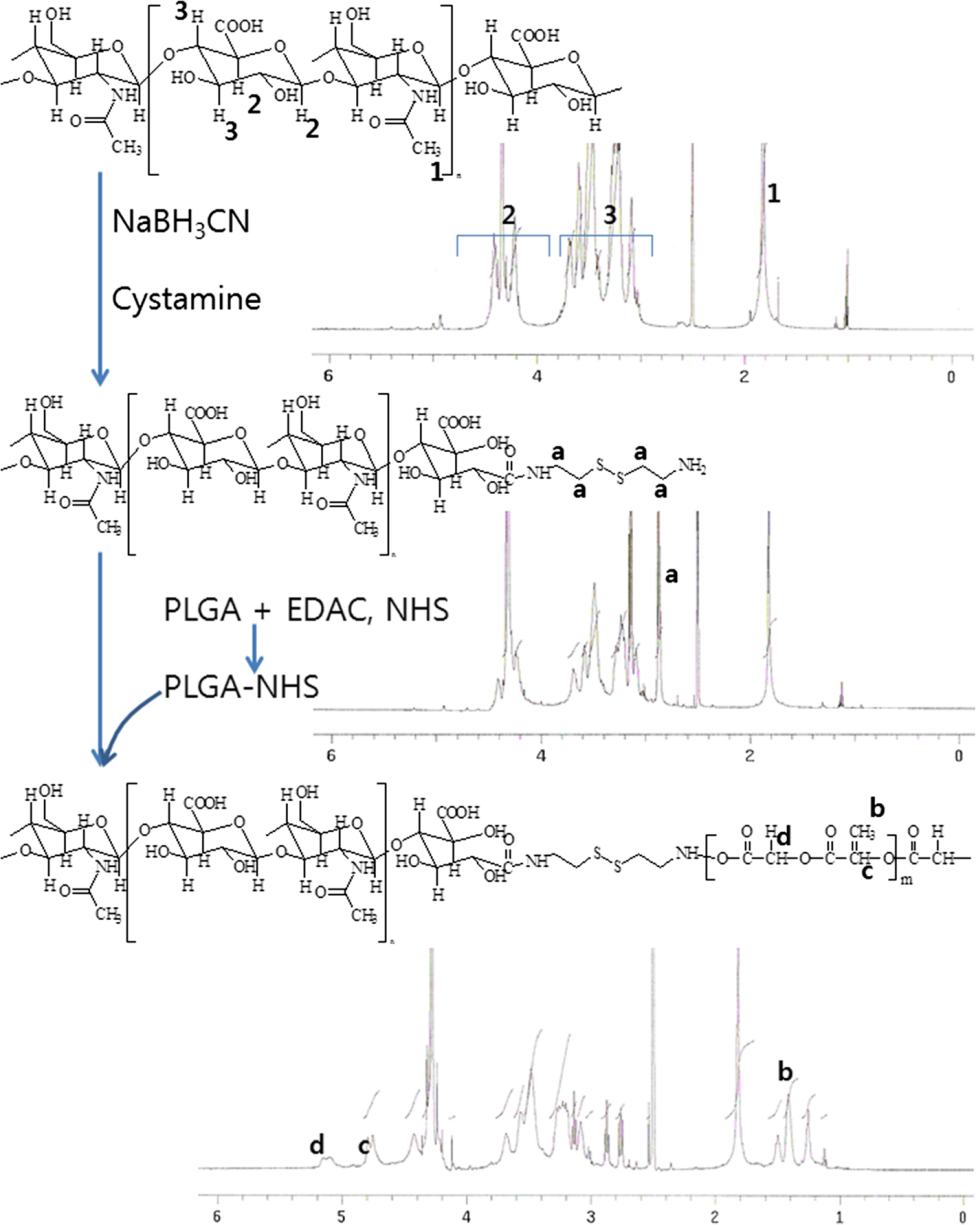
Synthesis scheme of HAssLG block copolymer

HA and PLGA were linked with cystamine linkage, and the block copolymer was synthesized as shown in Fig. 1. Prior to PLGA conjugation, cystamine was conjugated in the end of the HA chain by treatment with sodium cyanoborohydride because polysaccharides have one reductive end. To this, excess amount of cystamine was added to make aminated end of HA having disulfide linkage. Intrinsic peaks of HA were confirmed at 1.6~4.6 ppm at ^1^H NMR spectra. The ethylene proton of cystamine linkage was observed at 2.9 ppm as shown in Fig. 1. To the terminal amine of HA, PLGA was conjugated to construct the block copolymer having disulfide linkage. The specific proton peaks of PLGA were observed at 1.2~1.6 ppm and 4.6~5.2 ppm. The final yield of the HAssLG block copolymer was approximately 81 % (*w*/*w*).

### Characterization of Nanoparticles

**Fig. 2:**
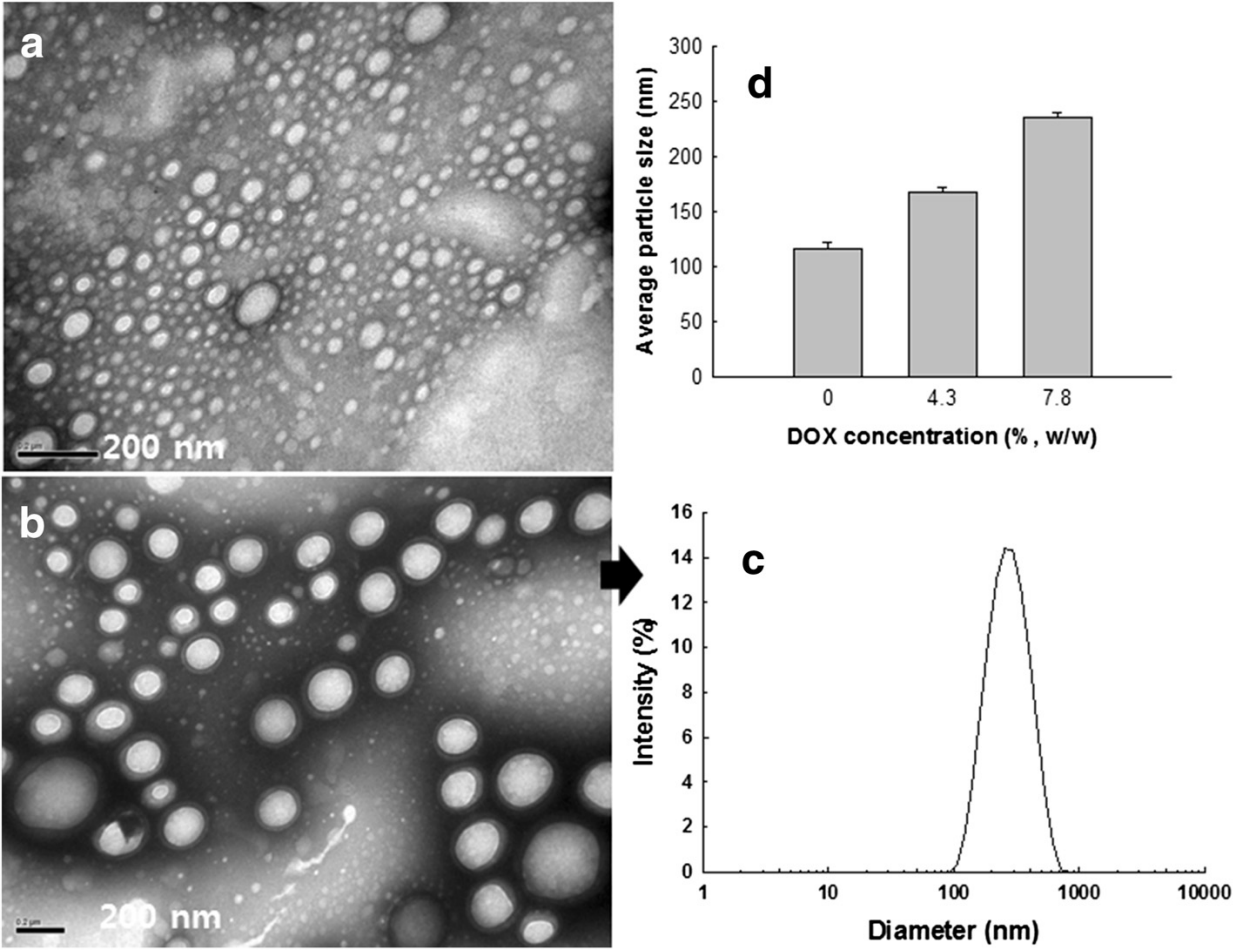
TEM photographs (**a**, **b**), particle size (**c**, **d**). TEM photographs of **a** empty HAssLG nanoparticles and **b** DOX-incorporated HAssLG nanoparticles (DOX contents, 7.8 % (*w*/*w*)). **c** Particle size distribution of DOX-incorporated HAssLG nanoparticles (DOX contents, 7.8 % (*w*/*w*)). **d** Particle size changes of HAssLG nanoparticles according to the increase of DOX contents

Generally, diblock copolymers composed of a hydrophilic domain and hydrophobic domain can form core-shell-type nanoparticles in the aqueous environment. Since HA is the hydrophilic one, it would form the outer shell of the nanoparticles while PLGA would form the core of the nanoparticles. Nanoparticles of the HAssLG block copolymer were fabricated in water by dialysis procedure. The morphology of empty HAssLG nanoparticles was a spherical shape as shown in Fig. 2a, and their sizes were less than 200 nm. Particle sizes were increased when DOX was incorporated into the nanoparticles as shown in Fig. 2b, d. DOX-loaded nanoparticles also have spherical shapes (Fig. 2b) and unimodal size distribution (Fig. 2c).

**Fig. 3:**
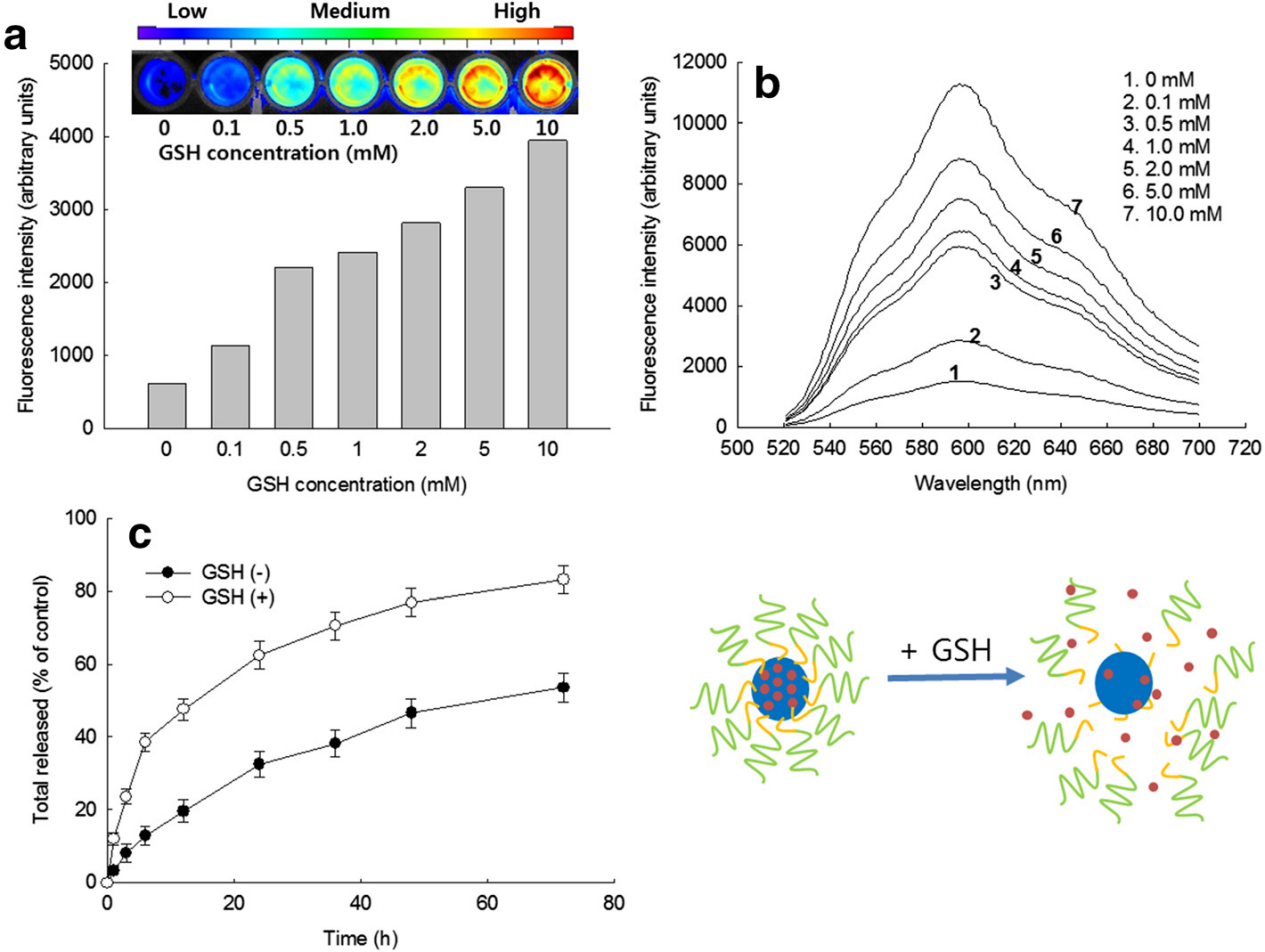
Fluorescence intensities (**a**) and fluorescence emission scan (**b**) of DOX-incorporated HAssLG nanoparticles according to the GSH concentration. DOX-incorporated nanoparticles were incubated 4 h at 37 °C in the presence of GSH. Fluorescence emission spectra were recorded in the range of 520~700 nm (excitation wavelength, 486 nm). Nanoparticle concentration was 1 mg/ml (DOX contents, 7.8 % (*w*/*w*)). **c** DOX release from HAssLG nanoparticles in the presence (GSH(+), 10 mM) or absence (GSH(−), 0 mM) of GSH

To study redox-response properties of nanoparticles, DOX-loaded nanoparticles were incubated at 37 °C with and without GSH as shown in Fig. 3a. DOX-loaded nanoparticles were treated with GSH for 4 h, and then fluorescence intensity of DOX was measured using a microplate reader and observed with imaging equipment as shown in Fig. 3a. As shown in Fig. 3a, fluorescence intensity rapidly increased according to the increase of GSH concentration. Furthermore, fluorescence emission spectra of DOX-loaded nanoparticles were recorded at 520~700 nm as shown in Fig. 3b. Fluorescence intensity was gradually increased according to the GSH concentration, indicating that DOX-incorporated HAssLG nanoparticles responded to GSH addition and then liberated DOX was controlled by GSH content. Furthermore, DOX release rate was increased when GSH was added to release media as shown in Fig. 3c. These results indicated that HAssLG nanoparticles have redox-responsive characters and delivery of anticancer drugs would be prompted according to the concentration of GSH.

### CD44 Receptor-Mediated Targeting of HAssLG Nanoparticles

**Fig. 4:**
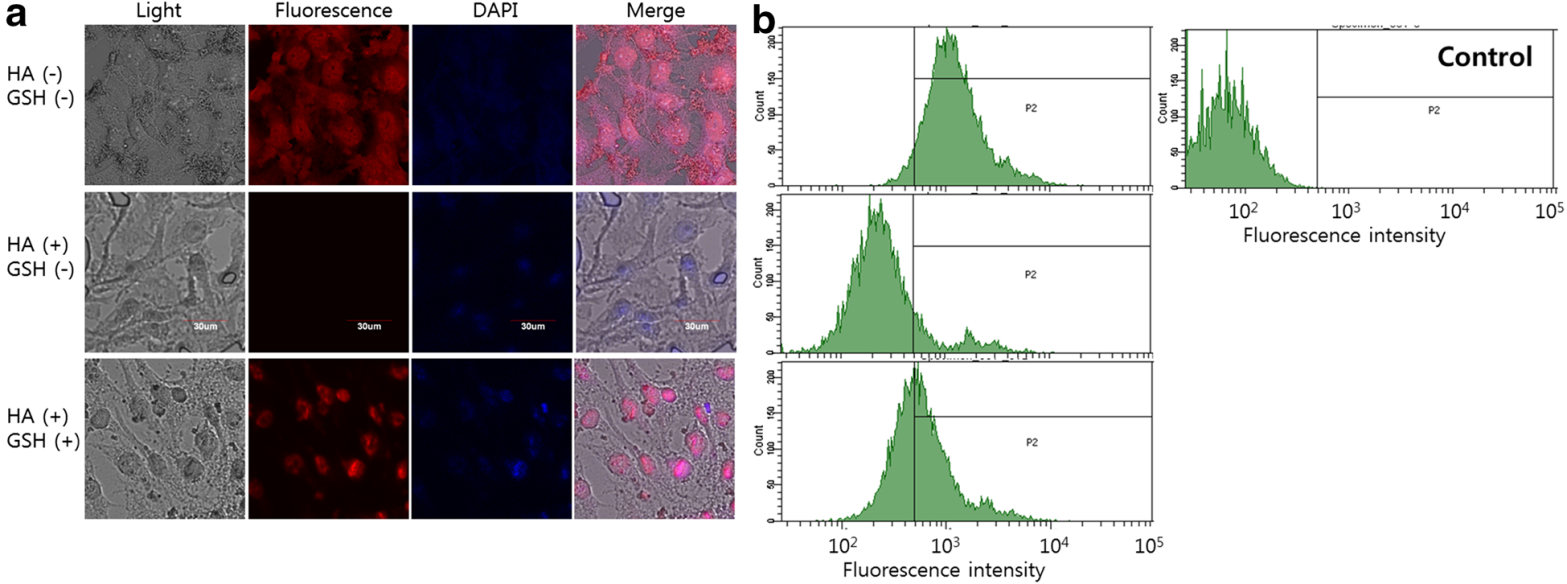
**a** Fluorescence images of MDA-MB231 cells. MDA-MB231 cells were exposed to DOX-incorporated HAssLG nanoparticles (DOX concentration, 5 μg/ml) for 1 h. Fluorescence images of cells were observed with CLSM. The CD44 receptor of MDA-MB231 cells was blocked with free HA (0.5 mg/ml) 1 h before treatment of DOX-incorporated nanoparticles. GSH (10mM) was added to study the effect of redox-responsive delivery of nanoparticles. **b** Flow cytometry analysis of MDA-MB231 cells

**Fig. 5:**
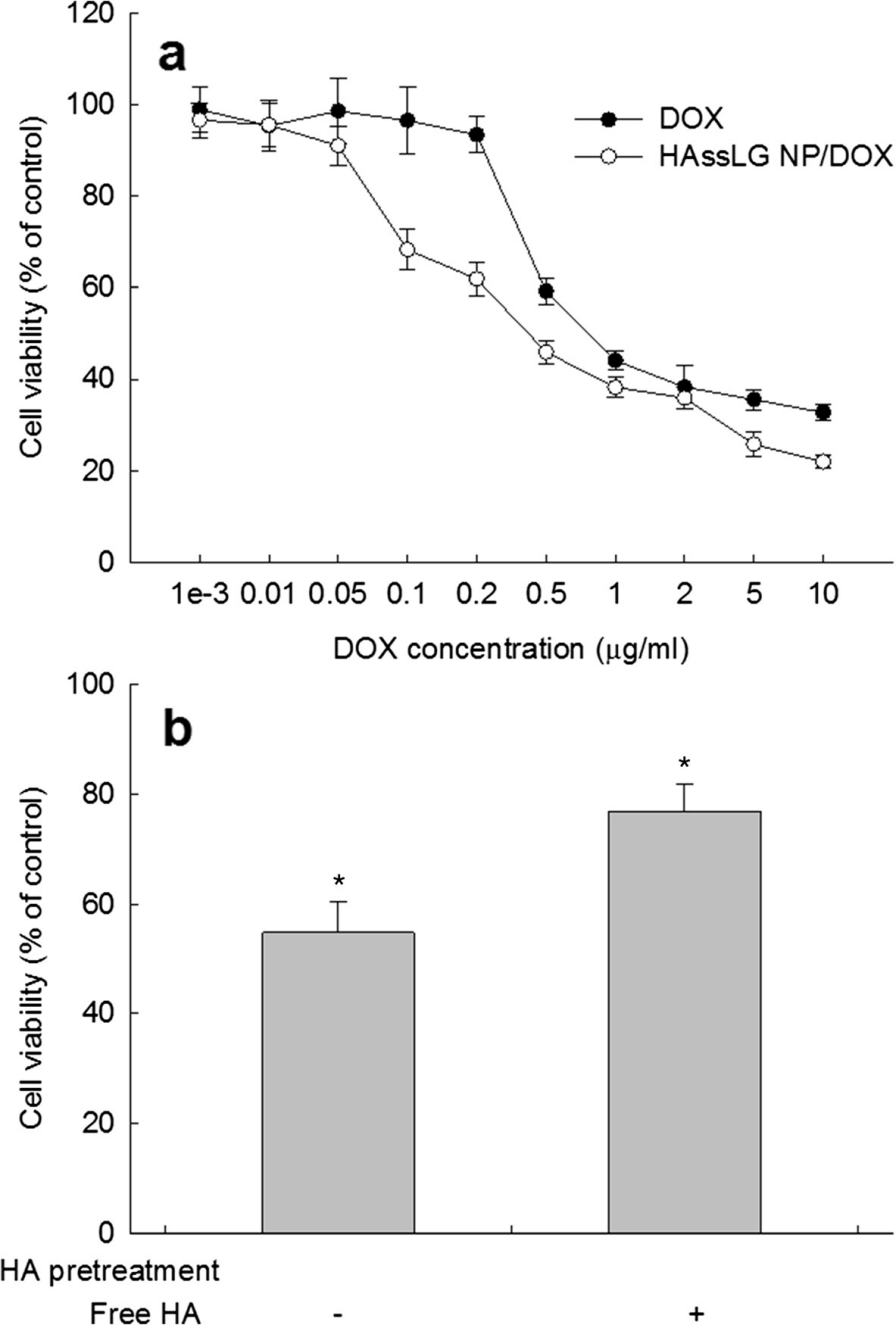
**a** Cytotoxicity of free DOX or DOX-incorporated HAssLG nanoparticles against MDA-MB231 cells. DOX or DOX-incorporated nanoparticles were exposed to cells 24h. **b**. Cytotoxicity of DOX-incorporated nanoparticles in the presence (+) or absence (-) of free HA against MDA-MB231 cells (*p < 0.05). MDA-MB231 cells were pretreated with free HA 1 h before drug treatment, and then cells were exposed to 50 μg/ml of DOX or nanoparticles for 4 h. Twenty-four hours later, viable cells were determined using the MTT assay

To investigate CD44-mediated delivery of HAssLG nanoparticles, CD44 receptor-overexpressed MDA-MB231 cells were employed. Since DOX itself expresses strong fluorescence intensity, DOX-loaded HAssLG nanoparticles were treated to MDA-MB231 cells as shown in Fig. 4a. As shown in Fig. 4a, red fluorescence of MDA-MB231 cells was strongly expressed when DOX-loaded nanoparticles were treated. However, fluorescence intensity was significantly decreased when the CD44 receptor was blocked by pretreatment with free HA as shown in Fig. 3a, indicating that HAssLG nanoparticles can target tumor cells through receptor-mediated endocytosis using the CD44 receptor. However, fluorescence intensity was slightly increased when GSH was added to the cell culture even though the CD44 receptor was blocked. These results indicate that HAssLG nanoparticles are responsive to both the CD44 receptor and redox environment. Furthermore, flow cytometry analysis of cells also supported these results, indicating that treatment of DOX-loaded HAssLG nanoparticles significantly increased fluorescence intensity as shown in Fig. 4b. Blocking of the CD44 receptor decreased fluorescence intensity of cells, and GSH addition increased fluorescence intensity of cells again. These results clearly supported Fig. 3a. Figure 5 shows the anticancer activity of nanoparticles against MDA-MB231 cells. As shown in Fig. 5, viability of MDA-MB231 cells was gradually decreased according to the increase of DOX concentration. Interestingly, HAssLG nanoparticles showed higher anticancer activity against MDA-MB231 cells, i.e., viability of cells with treatment of HAssLG nanoparticles was lower than that of DOX alone. Furthermore, cell viability with treatment of HAssLG nanoparticles was increased when free HA was pretreated as shown in Fig. 5b. These results indicated that HAssLG nanoparticles have enhanced anticancer activity with CD44 receptor targetability.

### In Vivo Animal Imaging Study

**Fig. 6:**
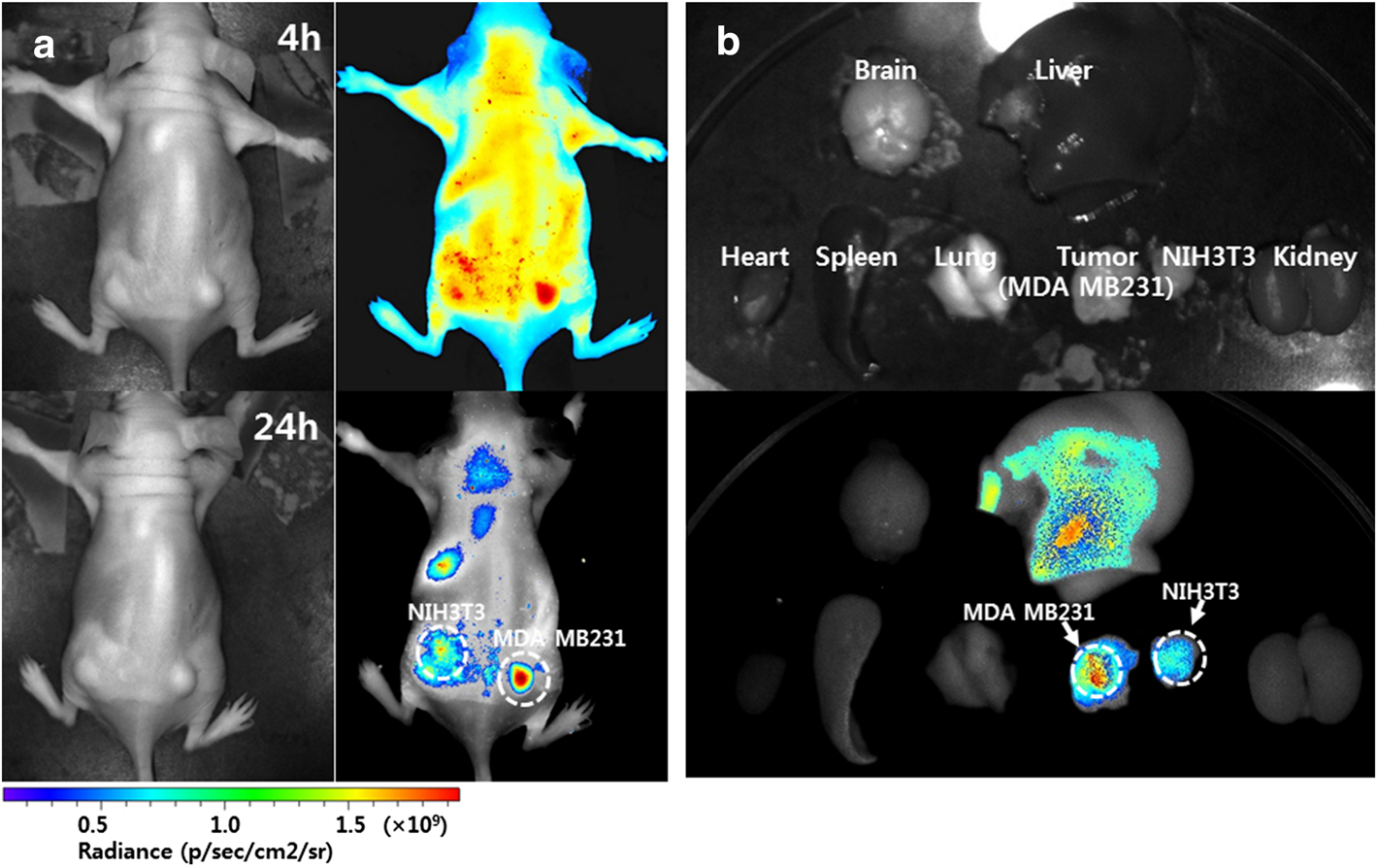
**a** Fluorescence imaging of MDA-MB231 cell-bearing (right flank) and NIH3T3 cell-bearing (left flank) mice. **b**. Organ-distribution of nanoparticles 24h later. 1 × 10^6^ MDA-MB231 cells and NIH3T3 cells were implanted into the right flank and left flank of BALb/C nude mice (5 weeks, 20~25 g), respectively. Three weeks later, NIR-dye-conjugated HAssLG nanoparticles (10 mg/kg) were intravenously injected via the tail vein. The major organs of the mice were taken 24 h after nanoparticle administration

To prove the CD44 receptor-mediated delivery capacity of HAssLG nanoparticles in vivo, CD44-positive MDA-MB231 cells and CD44-negative NIH3T3 cells were implanted into the right flank and left flank of the mice, respectively. NIR-dye-conjugated HAssLG nanoparticles were administered intravenously via the tail vein of mice, and distribution of nanoparticles in mice was observed with NIR imaging equipment as shown in Fig. 6. As shown in Fig. 6, fluorescence intensity of the MDA-MB231 tumor mass was significantly higher than that of the solid mass of NIH3T3. And then, these differences were increased 24 h after nanoparticle administration. As shown in Fig. 6b, fluorescence observation of each organ showed that the solid tumor of MDA-MB231 cells expressed stronger fluorescence intensity than that of NIH3T3 cells even though the liver also expressed strong fluorescence intensity. These results indicated that HAssLG nanoparticles have drug targeting potential against solid tumor via a specific pathway.

## Discussion

HA is a linear polysaccharide composed of d-glucuronic acid/*N*-acetyl-d-glucosamine units and has a vital role in the physiological phenomenon of cells such as adhesion, growth, migration, cell motility, inflammation, and invasion [31–36]. Especially, specific receptors for HA such as CD44 and RHAMM are frequently overexpressed in cancer cells rather than normal cells [11–18]. In addition to these findings, the fact that HA has biocompatibility, biodegradability, and functional moiety such as carboxylic acid stimulates application of HA in the biomedical field and drug targeting research [8, 19–25]. Hyung et al. reported that HA-decorated nanoparticles on the surface effectively targeted the CD44 receptor of CD44-overexpressed cells rather than lower CD44-expressed cells, while pure PLGA nanoparticles did not show targeting differences in both cells [7]. Since invasive cancer cells generally plentifully secreted hyaluronidase, anticancer agent release and delivery can be stimulated at invasive cancer cells and then specifically attack these cells through accelerated drug release by hyaluronidase [8].

Meanwhile, redox-responsive drug delivery has been also widely investigated for targeted drug delivery to tumor [26, 28, 29, 37]. Disulfide bond, which is abundant in the peptide and protein, can be cleaved by an intracellular reducing molecule, GSH, while it is stable at the extracellular environment [30]. Furthermore, the GSH level in the intracellular compartment (~10 mM) is known to be elevated compared to that in the extracellular environment (~10 μM). Especially, GSH level is elevated in the tumor cells [38]. These findings facilitated intracellular drug targeting strategy by the introduction of disulfide bond in the polymer chain [27, 37]. Sun et al. synthesized a diblock copolymer having a disulfide bond between dextran and poly(ε-caprolactone), and then drug release rate was increased through cleavage of disulfide bond by GSH [37]. These phenomena enabled nanovehicles to target the intracellular compartment.

**Fig. 7:**
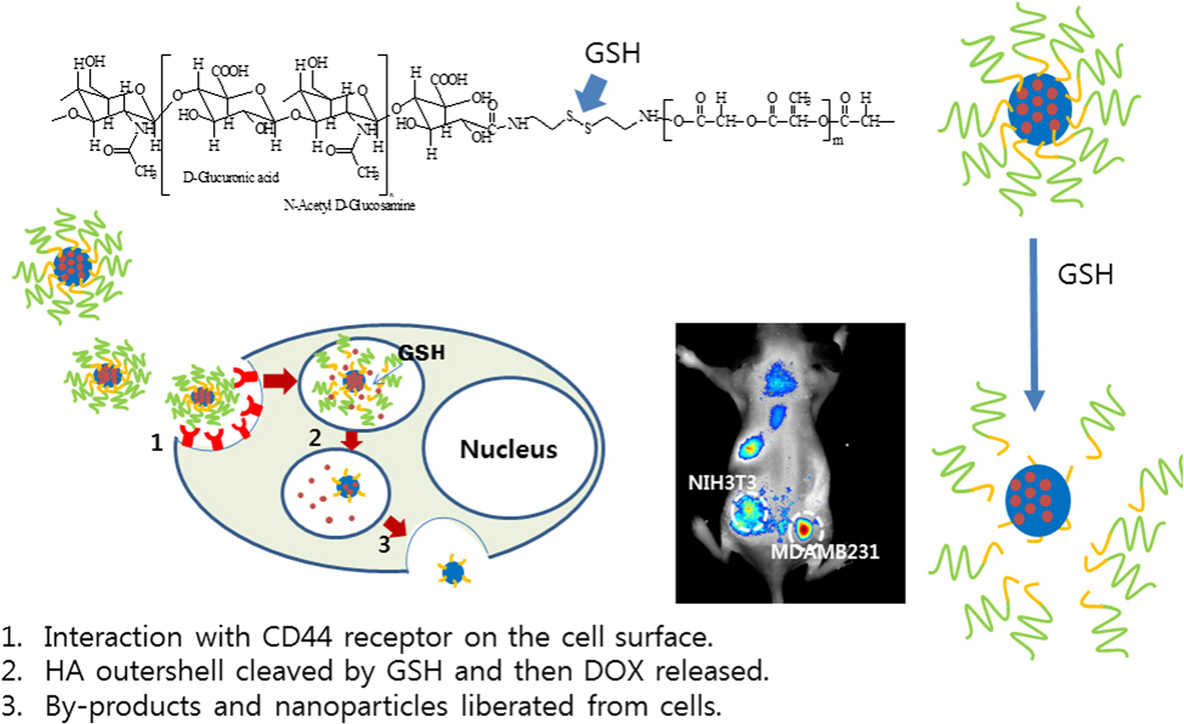
Schematic illustration of tumor cell targeting using HAssLG nanoparticles

Our concept is to attain sequential targeting of tumor cells as shown in Fig. 7, i.e., nanoparticles are accumulated in the tumor tissue by the EPR effect and then they target tumor cells by specific interaction with the CD44 receptor on the tumor cell membrane followed by receptor-mediated endocytosis of them. Then, GSH in the cytosol cleaved the HA outer shell of HAssLG nanoparticles, and DOX release would be facilitated in the intracellular environment. This hypothesis was proved by fluorescence intensity measurement of DOX-loaded HAssLG nanoparticles in the presence of GSH as shown in Fig. 3a, b. Fluorescence intensity of DOX was dose-dependently increased by GSH, indicating that GSH cleaved the HA outer shell of the nanoparticles and then drug release rate was promoted as shown in Fig. 3c. Furthermore, CD44 receptor-specific endocytosis of nanoparticles was observed and, otherwise, CD44 receptor blocking is to interrupt cellular uptake of nanoparticles as shown in Fig. 4a. Furthermore, DOX delivery into the tumor cells was increased again by the addition of GSH even though the CD44 receptor of tumor cells was blocked and nanoparticle uptake was inhibited, indicating that DOX release was facilitated by GSH addition and subsequent cleavage of the HA outer shell. CD44 receptor specificity of HAssLG nanoparticles was also proved by an in vivo animal NIR fluorescence imaging study as shown in Fig. 6. To prove the specificity of HAssLG nanoparticles, CD44-positive MDA-MB231 cells and CD44-negative NIH3T3 mouse fibroblast cells were simultaneously implanted into the right flank and left flank of the mice, respectively, and then the solid tumor was prepared. As shown in Fig. 6, NIR fluorescence intensity in the back of mice was significantly higher at tumor mass of MDA-MB231 than at solid mass of NIH3T3, indicating that HAssLG nanoparticles specifically targeted MDA-MB231 cells in vivo and they would deliver the drug to tumor cells in a specific manner.

## Conclusions

A HAssLG diblock copolymer was synthesized for application in the sequential targeting concept of nanoparticles by CD44 receptor-mediated and redox-responsive drug delivery. HAssLG nanoparticles have spherical shapes with nanosized diameter. DOX-loaded HAssLG nanoparticles were prepared by dialysis procedure. DOX release was facilitated by addition of GSH, indicating that the disulfide bond of HAssLG nanoparticles was cleaved and then DOX release rate was increased. In an in vitro cell culture study, DOX-loaded HAssLG nanoparticles were endocytosed by CD44 receptor-mediated pathway. An animal NIRF imaging study also proved that HAssLG nanoparticles were delivered to tumor tissues through a targeting mechanism. We suggest that HAssLG nanoparticles are a promising candidate for tumor targeting.
